# *Ashea megacephala* Kim & Ahn (Coleoptera, Staphylinidae, Aleocharinae), a new gyrophaenine genus and species from Peru

**DOI:** 10.3897/zookeys.530.6110

**Published:** 2015-10-28

**Authors:** Yoon-Ho Kim, Kee-Jeong Ahn

**Affiliations:** 1 Scientific Collection & Conservation Team, National Science Museum, Daejeon 34143, Republic of Korea; 2 Department of Biology, Chungnam National University, Daejeon 34134, Republic of Korea

**Keywords:** Gyrophaenina, key, new genus, new species, Peru, Staphylinidae

## Abstract

*Ashea megacephala*, a new Peruvian genus and species is described. The specimens were collected on mushrooms and mushroom-associated trees. This genus can be readily distinguished from the other genera of the subtribe Gyrophaenina by the large head and the three indistinctly articulated labial palpomeres. A key to the known genera of Gyrophaenina is provided. A habitus photograph and illustrations of diagnostic characters are also presented.

## Introduction

The subtribe Gyrophaenina Kraatz contains 833 species in 21 genera worldwide (Newton and Thayer 2005, Ashe 2007) and most are obligate inhabitants of fresh mushrooms in the larval and adult stages (Ashe 1984). Members of Gyrophaenina are characterized by the following characters: lacinia truncate at apex with well-developed spinose area, spines and setae reduced on inner margin of lacinia; labial palpus with two palpomeres, non-styliform, one medial seta on prementum; mesocoxal cavities broadly separated, broad meso- and metaventral processes not joined by isthmus but meeting along broad suture (Ashe 2001).

During an ongoing worldwide revisionary study of the Homalotini, a series of small specimens were found in the Snow Entomological Museum Collection, Lawrence, USA, each with very large head. After detailed study of the specimens, we conclude that this is a new genus and species belonging to Gyrophaenina.

In this paper, *Ashea megacephala* gen. n. and sp. n. is described and a key to the known genera of Gyrophaenina is provided, as well as a habitus photograph with illustrations of diagnostic characters.

## Methods

Specimens were dissected in water and mounted on sticky carbon tape. They were dried at 60 °C on a slide warmer for 24 h, sputter-coated with Pt/Pd nanoparticles using a sputter coater (208 HR, Cressington Scientific Instruments, Watford, Hertfordshire, UK), and examined with SEM (S-4800, Hitachi High-Technologies, Tokyo, Japan). Descriptive terms used here follow Ashe (1984). Holotype and six paratypes are deposited in Snow Entomological Museum Collection (SEMC), University of Kansas, Lawrence, USA. Six paratypes are deposited in the Chungnam National University Insect Collection (CNUIC), Daejeon, Korea.

## Results

### Ashea gen. n.

Taxon classificationAnimaliaColeopteraStaphylinidae

http://zoobank.org/69A1EB75-782C-4E89-BEA6-CABBE067AF8F

#### Type species.

*Ashea megacephala* sp. n.

#### Diagnosis.

Head (Fig. 2) very large, as wide as and distinctly longer than pronotum; eye large, longer than temple; labrum (Fig. 4) markedly transverse, seven pairs of macrosetae present; right mandible (Fig. 5) with very large median tooth; ligula (Fig. 14) short, entire apically, labial palpus with three indistinct palpomeres; pronotum (Fig. 8) markedly transverse, more than 2.0 times as wide as long; hypomeron not visible in lateral aspect; mesoventrite (Fig. 15) without medial longitudinal carina; tergite X (Fig. 9) with medial setose area arranged in distinct V-shape, composed of two indistinct rows of setae, setae subspatulate; median lobe (Fig. 10) bulbous at base, apical process long and slender.

#### Description.

**Figures 1.**
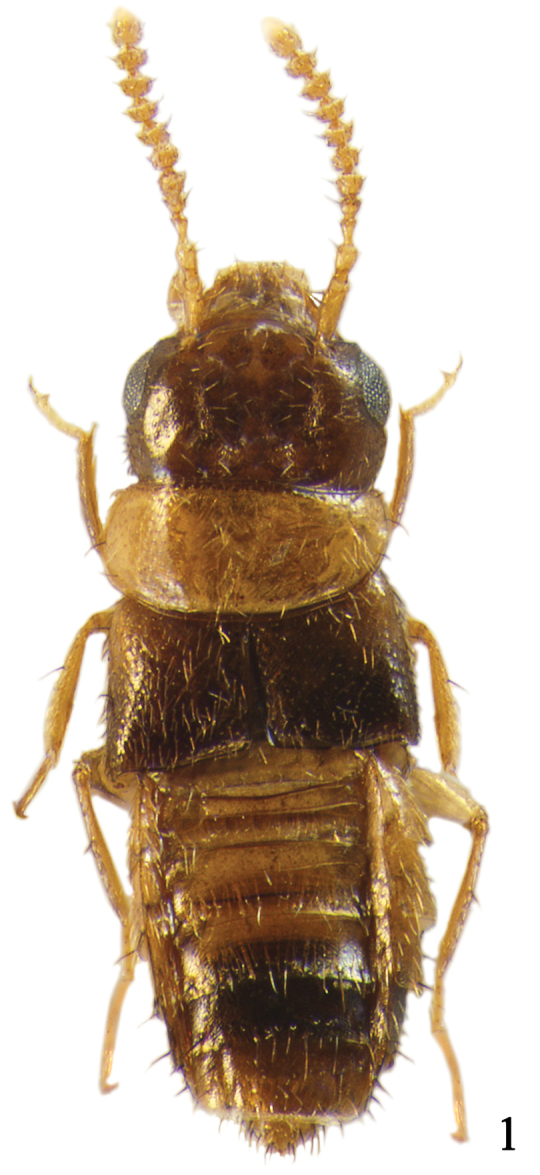
*Ashea megacephala*, habitus, 1.3 mm.

**Figures 2–7.**
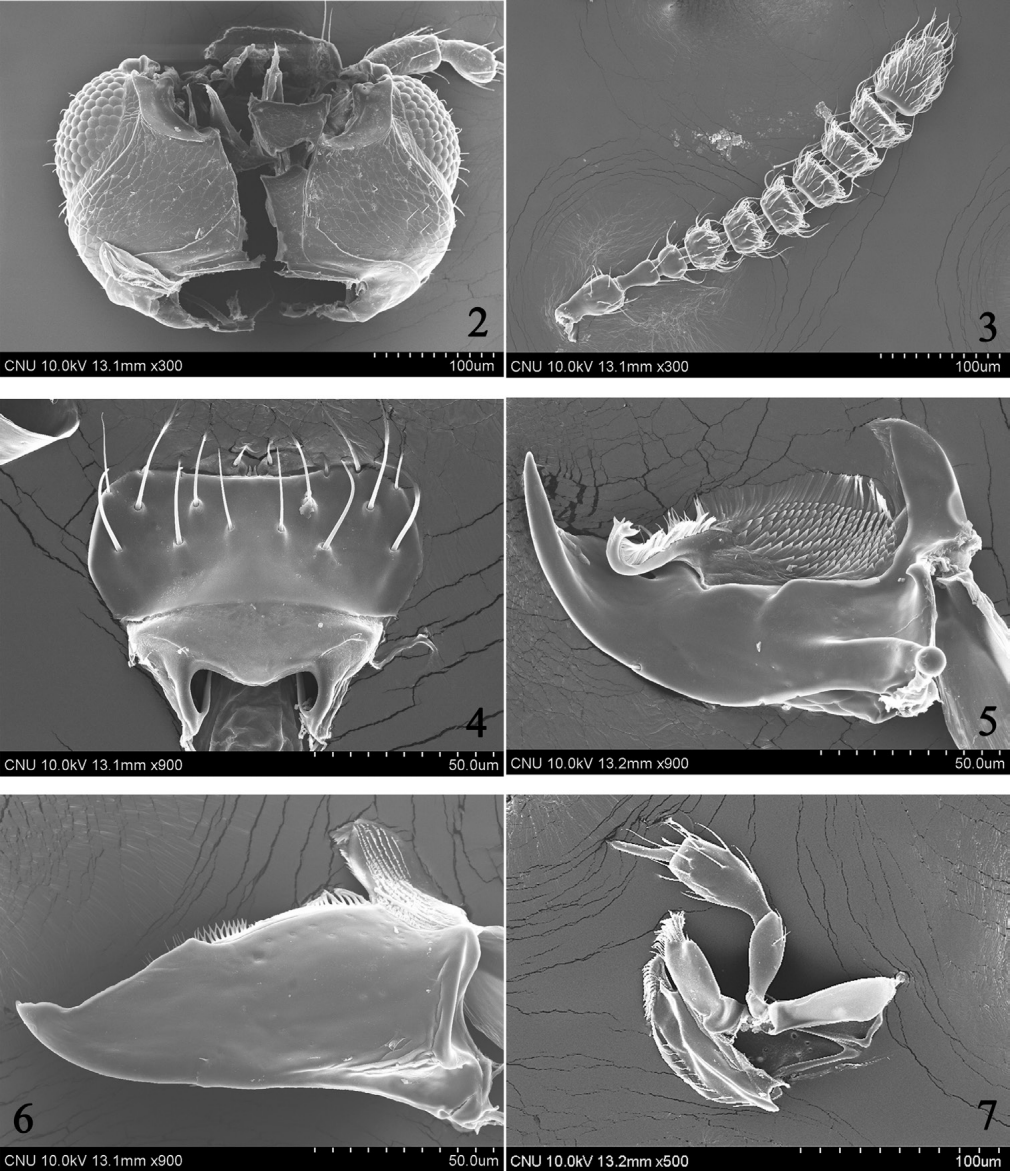
*Ashea megacephala*, SEM photographs: **2** head, ventral aspect **3** antenna (antennomeres 2–11) **4** labrum, ventral aspect **5** right mandible, ventral aspect **6** left mandible, dorsal aspect **7** maxilla, ventral aspect.

**Figures 8–13.**
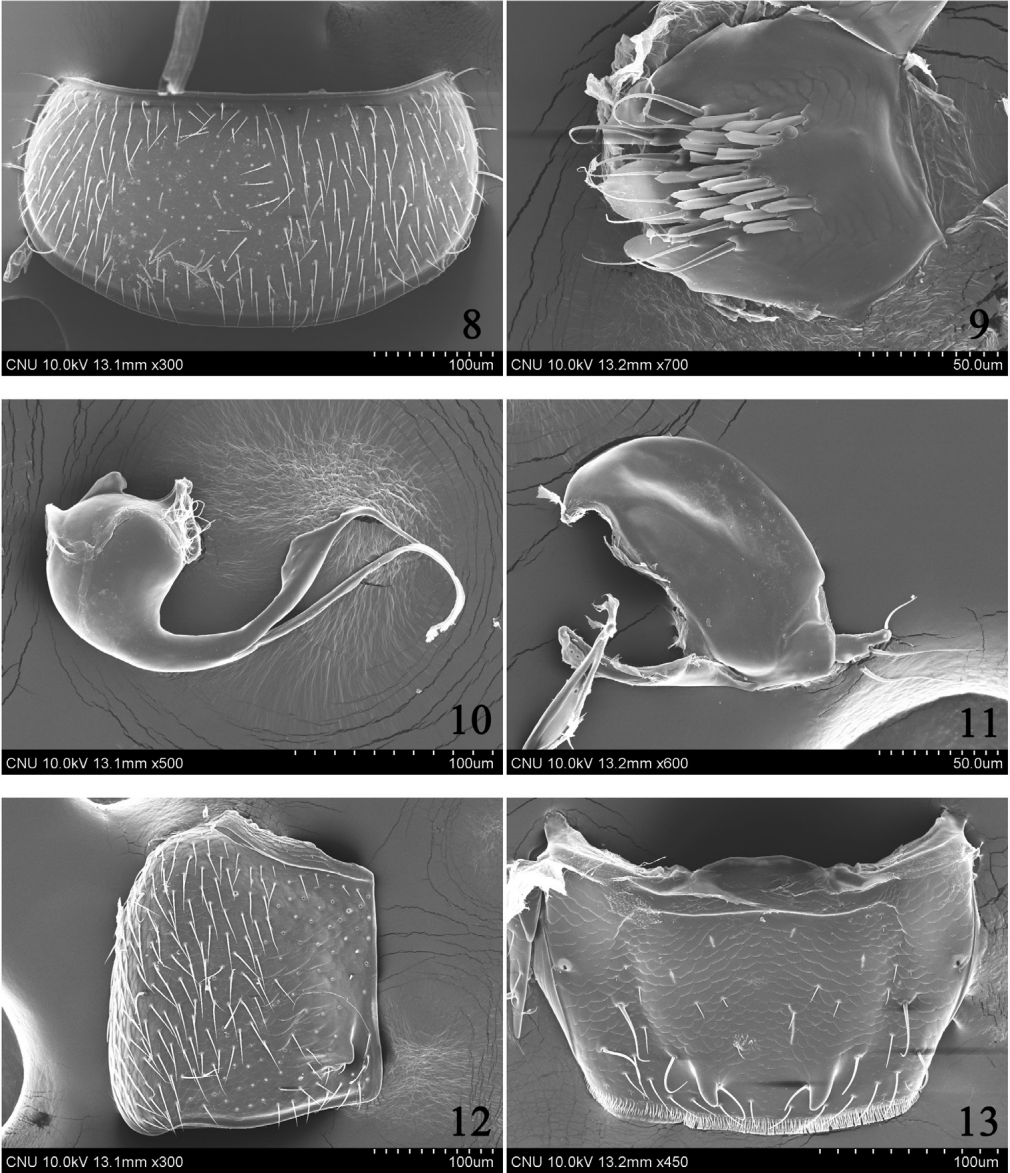
*Ashea megacephala*, SEM photographs: **8** pronotum, dorsal aspect **9** tergite X, dorsal aspect **10** median lobe, lateral aspect **11** paramere, lateral aspect **12** male elytron, dorsal aspect **13** male tergite VII, dorsal aspect.

**Figures 14–17.**
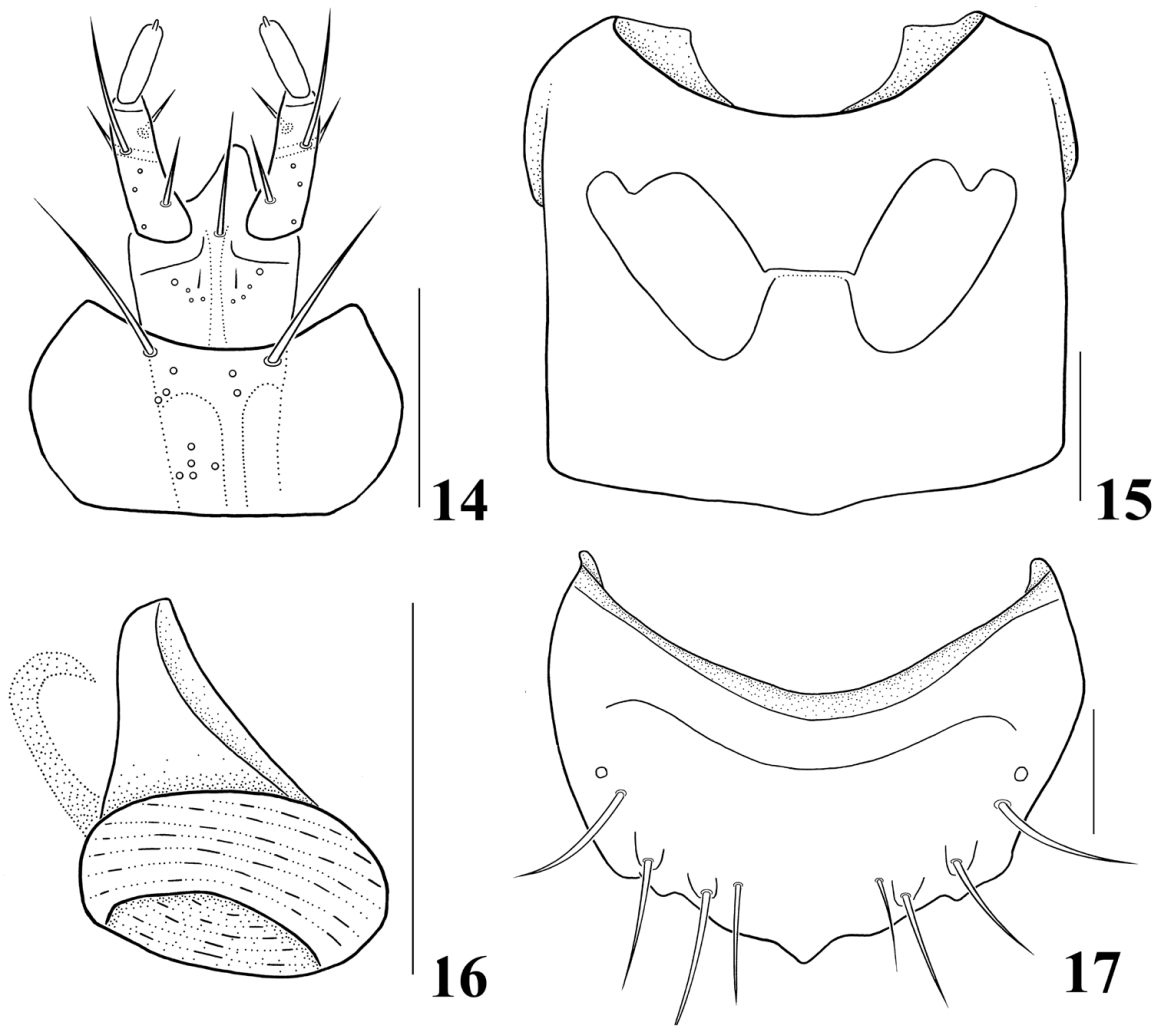
*Ashea megacephala*: **14** labium, ventral aspect **15** meso- and metaventrites, ventral aspect **16** spermatheca, **17** male tergite VIII, dorsal aspect. Scale bars 0.1 mm.

Body (Fig. 1) very small, length 1.0–1.4 mm. Body slightly flattened dorso-ventrally, parallel-sided; surface sculpture reticulate throughout, slightly glossy and pubescent; light brown to brown but head, elytra, posterior half of abdominal tergite V and tergites VI–VII dark brown. *Head.* (Figs 1–2) Very large, slightly transverse and flattened, as wide as and distinctly longer than pronotum; eye large, longer than temple; infraorbital carina well developed, complete; gular suture moderately separated, subparallel-sided; antenna (Fig. 3) moderate in size, with eleven antennomeres, antennomere 4 transverse, 5–7 slightly transverse, 8–10 transverse, 5–10 slightly increase in relative width from base to apex. *Mouthparts.* Labrum (Fig. 4) markedly transverse, seven pairs of macrosetae present, sensilla of antero-medial sensory area distinct, α-sensillum with short setose process, β and γ minute and conical, ε with short setose process, almost as long as α, two lateral sensilla present on lateral margin of epipharynx, without transverse row of sensory pores on basal region of epipharynx; mandible (Figs 5–6) asymmetrical, decurved and pointed apically, ventral condylar molar patch moderate in size with densely arranged denticles, prostheca well developed, right mandible with very large median tooth; maxilla (Fig. 7) with four palpomeres, palpomeres 2–3 slightly dilated distally, 3 longer than 2, 4 elongate with a small spine at apex, filamentous sensilla reaching to basal third, lacinia with apex obliquely truncate with well developed “spore brush”, teeth of spore brush small and densely arranged, inner margin without spines, with a longitudinal row of setae; labium (Fig. 14) with ligula short, entire apically, labial palpus indistinctly composed of three palpomeres, palpomere 1 almost as long as 3, distinctly longer than 2, one medial seta present on prementum, medial pseudopore field of prementum narrow and without pseudopores, lateral pseudopore field with one setose pore, one real pore and three pseudopores, mentum moderately emarginate in anterior margin. *Thorax.* Pronotum (Fig. 8) markedly transverse, more than 2.0 times as wide as long, widest in middle, surface slightly pubescent, several macrosetae present; hypomeron not visible in lateral aspect; prosternum transverse, without distinct median knob; elytra (Fig. 12) slightly wider and distinctly longer than pronotum, postero-lateral margin moderately sinuate; hind wing without setose lobe on flabellum; mesoventrite (Fig. 15) without medial longitudinal carina, mesoventral process broad and not fused to metaventral process, apex truncate; apex of metaventral process indistinguishable; isthmus absent; mesocoxal cavities widely separated; metepisternum with single row of setae; tarsal formula 4-4-5, tarsomere 1 of pro– and mesolegs as long as 2, 1 of metaleg slightly longer than 2, with one empodial seta between tarsal claws, shorter than claw. *Abdomen.* Tergites III–VI transversely impressed; tergite X (Fig. 9) with medial setose area arranged in distinct V-shape, composed of two indistinct rows of setae, setae subspatulate, with six to nine macrosetae on each side of midline. *Genitalia.* Spermatheca (Fig. 16) simple and round; median lobe (Fig. 10) bulbous at base, apical process long and slender, flagellum long, slender and more or less whip-like; paramere (Fig. 11) with apical lobe of paramerite short and subcylindrical with four setae, paramerite enlarged, slightly longer than apex of condylite. *Secondary sexual characteristics.* Male: elytron (Fig. 12) with tubercle near suture about 0.2 length of elytron from posterior margin; subapical margin of tergite VII (Fig. 13) with two tooth-like tubercles; posterior-median margin of tergite VIII (Fig. 17) with triangular projection.

#### Etymology.

Named after the late James S. Ashe in honor of his research on the subtribe Gyrophaenina. He was the first collector of these beetles.

#### Distribution.

Peru.

#### Remarks.

*Ashea* gen. n. can be distinguished from other gyrophaenine genera by the diagnostic characters presented above. Especially, the combination of the large head and indistinctly articulated three labial palpomeres clearly makes this new genus unique among all other Gyrophaenina.

*Ashea* gen. n. belongs to the “*Gyrophaena*” lineage (*sensu* Ashe 1984) based on the following characters: body slightly pubescent; ligula entire apically, prementum with a single medial seta. Among the lineage, this genus is probably the most closely related to *Eumicrota* Casey. These two genera share a small body size, entire and protruded ligula, markedly transverse pronotum, mesoventrite without medial longitudinal carina, tergite X with medial setose area arranged in distinct V-shape, and median lobe with apical process slender and elongate.

Tergite X provides useful characters in the study of the subtribe Gyrophaenina classification (Ashe 1984). Loss of setae antero-medially and postero-laterally results in one or a few rows of setae arranged in a distinct “V” shape. This distribution of microsetae is found only in *Eumicrota* and *Ashea* gen. n.

On the other hand, the genus *Ashea* gen. n. differs from *Eumicrota* by the following diagnostic characters: head very large, as wide as and distinctly longer than pronotum; right mandible with very large median tooth; labial palpus with three indistinctly separated palpomeres.

We have not had the opportunity to study any specimens of the following three gyrophaenine genera (*Brachycantharus* Bierig, *Microbrachida* Bierig, *Neobrachychara* Bierig) described from Central America by Bierig (1939). Original descriptions of these genera did not include sufficient diagnostic characters and they have not been redescribed since their original description. However, Bierig (1939) provided very clear scientific habitus illustrations and they can be easily distinguished from *Ashea* gen. n. by the following diagnostic characters: body subparallel-sided in *Ashea* (body elongated-oval, sides of abdomen convergent to apex in *Microbrachida*); pronotum strongly transverse in *Ashea* (pronotum moderately transverse in *Brachycantharus*); tergite X with medial setose area arranged in distinct V-shape in *Ashea* (tergite X with medial setose area arranged in chevron-shape in *Neobrachychara*).

### Ashea megacephala

Taxon classificationAnimaliaColeopteraStaphylinidae

Kim & Ahn sp. n.

http://zoobank.org/BC7C5FB9-EF97-45F7-9571-DA6856CEC74A

#### Type material.

**Holotype**, male, labeled as follows: Peru: Tambopata Prov., 15 km NE Pto. Maldonado, 6 July 1989, 200 m, J. Ashe, R. Leschen, #427, *ex: Daedaleopsis*; Holotype, *Ashea megacephala* Kim and Ahn, Desig. Y.-H. Kim and K.-J. Ahn 2015. **Paratypes**, 5♂♂4♀♀ (2♂♂1♀ on slides), same data as holotype; 1♀, Tambopata Prov., 15 km NE Pto. Maldonaldo, 13 July 1989, 200 m, J. Ashe, R. Leschen, #507, *ex*: rotten tree; 1♀, Tambopata Prov., 15 km NE Pto. Maldonado, 13 July 1989, 200 m, J. Ashe, R. Leschen, #508, *ex*: polypore; 1♀, Tambopata Prov., 15 km NE Pto. Maldonado, 13 July 1989, 200 m, J. Ashe, R. Leschen, #515, *ex: Irpex*-like; 1♀, Tambopata Prov., 15 km NE Pto. Maldonado, 9 July 1989, 200 m, J. Ashe, R. Leschen, #454, *ex: Schizopora*; 2♀♀, Tambopata Prov., 15 km NE Pto. Maldonado, 17 July 1989, 200 m, J. Ashe, R. Leschen, #537, *ex: Thelephoraceae*; 1♀, Tambopata Prov., 15 km NE Pto. Maldonado, 16 July 1989, 200 m, J. Ashe, R. Leschen, #86, *ex: Auricularia auricula*.

#### Description.

Body length 1.0–1.4 mm. *Head.* Very large, slightly transverse and flattened, as wide as and distinctly longer than pronotum; eye large, longer than temple, length ratio of eye to temple 1.56; antennomere 1 longest, about 2.8 times as long as wide, 2 about 1.5 times as long as wide and 1.5 times as long as 3, 3 about 1.4 times as long as wide, 4 about 1.4 times as wide as long, 5 about 1.1 times as wide as long, 6–7 about 1.2 times as wide as long, 8–9 about 1.3 times as wide as long, 10 about 1.36 times as wide as long, 11 about 1.4 times as long as wide (Fig. 3). *Mouthparts.* Labrum (Fig. 4) markedly transverse, about 2.3 times as wide as long; mandible (Figs 5–6) about 1.5 times as long as basal width, ventral condylar molar patch moderate in size, about 0.3 times of basal width; maxillary palpomere 2 (Fig. 7) about 2.3 times as long as wide, 3 about 2.25 times as long as wide and about 1.4 times as long as 2; prementum (Fig. 14) with medial seta distinctly longer than ligula. *Thorax.* Pronotum (Fig. 8) markedly transverse, more than 2.0 times as wide as long, widest at middle; mesoventral process (Fig. 15) extended to about half of mesocoxal cavities. *Genitalia.* Spermatheca (Fig. 16) simple and round at base; median lobe (Fig. 10) bulbous at base, apical process long, slender and recurved subapically, flagellum long, slender and more or less whip-like; paramere (Fig. 11) with apical lobe of paramerite short and subcylindrical with four setae, basal two distinctly longer than others, paramerite enlarged, slightly longer than apex of condylite. *Secondary sexual characteristics.* Male: elytron (Fig. 12) with tubercle at near suture about 0.2 length of elytron from posterior margin; subapical margin of tergite VII (Fig. 13) with two tooth-like tubercles; posterior-medial margin of tergite VIII (Fig. 17) with triangular projection.

#### Distribution.

Tambopata, Peru.

#### Etymology.

Named from the Greek *mega* meaning “large” and *cephalus* meaning “head”, which refers to large head.

#### Remarks.

Specimens were collected on mushrooms and/or mushroom associated trees in Peru.

### Key to the genera of the “*Gyrophaena*” lineage of subtribe Gyrophaenina Kraatz (modified from Ashe 1984)

**Table:** 

1	Pronotum (Fig. 8) markedly transverse, about 2.0 times as wide as long; tergite X (Fig. 9) with medial setose area in distinct V-shaped row	**2**
–	Pronotum (Kim and Ahn 2014: Fig. 7H) of most specimens 1.2 to 1.7 times as wide as long; tergite X with medial setose area more or less subquadrate	**3**
2	Head (Fig. 1) large, distinctly longer than pronotum; right mandible with large median tooth; labial palpus with three indistinct palpomeres	***Ashea* gen. n.**
–	Head moderate in size, shorter than or as long as pronotum; right mandible with small median tooth; labial palpus with two palpomeres	***Eumicrota* Casey**
3	Eyes extremely large (Kim and Ahn 2014: Fig. 8A), occupying most of lateral margins of head	***Phanerota* Casey**
–	Eyes moderate in size (Kim and Ahn 2014: Fig. 8B)	***Gyrophaena* Mannerheim**

## Supplementary Material

XML Treatment for Ashea

XML Treatment for Ashea megacephala
